# Duodenal α-Synuclein PFF Injection Induces Vagus-Related Gut-to-Brain Pathology in C57BL/6J and A53T Transgenic Mice

**DOI:** 10.3390/brainsci16080804

**Published:** 2026-07-30

**Authors:** Mengfei Wang, Guangqiang Sun, Peifeng Wan, Zitong Wang, Yali Nie, Hongchun Liu, Meiyu Geng, Ming Liu, Yu Zhang

**Affiliations:** 1 Key Laboratory of Marine Drugs, Ministry of Education, School of Medicine and Pharmacy, Ocean University of China, Qingdao 266003, China; 2 Shandong Laboratory of Yantai Drug Discovery, Bohai Rim Advanced Research Institute for Drug Discovery, Yantai 264117, China; 3 State Key Laboratory of Drug Research, Shanghai Institute of Materia Medica, Chinese Academy of Sciences, Shanghai 201203, China

**Keywords:** Parkinson’s disease, Braak hypothesis, α-Synuclein, preformed fibrils, vagus nerve, gut microbiota, A53T

## Abstract

**Highlights:**

**What are the main findings?**

- Duodenal α-syn PFF injection induces ascending pathology: Injection of α-syn preformed fibrils (PFF) into the duodenal muscularis led to phosphorylated α-syn (p-α-syn) pathology propagating to the brain in both C57BL/6J and A53T transgenic mice.
- Truncal vagotomy was associated with attenuation of gut-to-brain propagation of p-α-syn, reduced dopaminergic neuron loss, improved motor function, and alterations in the gut microbiome.

**What are the implications of the main findings?**

- Experimental validation of the Braak hypothesis: These findings are consistent with the hypothesis that pathological α-syn spread from the gut to the brain is related to vagus nerve signaling, supporting the Braak hypothesis for Parkinson’s disease initiation.
- A useful gut-origin PD model: This study provides a mouse model that recapitulates key PD features, offering a tool for investigating gut–brain axis mechanisms and potential therapies.

**Abstract:**

**Background**: The Braak hypothesis proposes that α-synuclein (α-syn) pathology may originate in the gastrointestinal tract and propagate to the central nervous system along the gut–brain axis; however, the precise propagation routes and the factors influencing this process remain controversial. **Methods**: A gut-originating Parkinson’s disease model was established by injecting α-syn preformed fibrils (PFF) into the duodenal muscularis of C57BL/6J and A53T transgenic mice. Phosphorylated α-synuclein (p-α-syn) pathology, motor behavior, and gut microbiota were assessed, with truncal vagotomy included to evaluate its association with gut-to-brain propagation. **Results**: In C57BL/6J mice, at 4 months post-injection, p-α-syn deposition was observed in both the duodenal muscular layer and the striatum, accompanied by gut microbiota alterations and motor behavioral deficits. Truncal vagotomy was associated with reduced p-α-syn levels in the brain and alterations in the gut microbiota. In A53T transgenic mice, p-α-syn pathology and neurodegenerative changes were also observed following α-syn PFF injection, though the lack of a genetically matched wild-type control precludes definitive attribution of these phenotypes solely to the A53T transgene. **Conclusions**: These results align with the Braak hypothesis, showing that gut-derived p-α-syn pathology and associated functional impairments are intimately linked to vagal pathways during their propagation to the brain. Additionally, this gut-origin PD mouse model may serve as a useful tool for future mechanistic investigations.

## 1. Introduction

Parkinson’s disease (PD) is the second most common neurodegenerative disorder worldwide and represents a prototypical α-synuclein (α-syn) aggregation-associated neurodegenerative disease [1]. The pathological hallmarks of PD include progressive loss of nigrostriatal dopaminergic neurons and the formation of intraneuronal Lewy bodies (LBs), which are primarily composed of aggregated α-syn [2]. In the brains of patients with PD, widespread phosphorylation of α-syn at serine 129 (Ser129) occurs concomitantly with LBs formation and dopaminergic neuronal loss. Approximately 90% of α-syn deposited within LBs is phosphorylated at Ser129, whereas in non-PD individuals only 4% or less of α-syn is phosphorylated at this residue [3,4]. These observations indicate that Ser129 phosphorylation of α-syn is tightly regulated under physiological conditions and represents a key molecular marker of pathological aggregation [5].

In contrast to the traditional view that PD originates in the brain, the Braak hypothesis proposes that pathological α-syn may originate in the gastrointestinal tract and propagate along the vagus nerve, sequentially involving vulnerable regions of the medulla oblongata, pontine tegmentum, midbrain, basal forebrain, and ultimately the cerebral cortex [6,7,8]. Previous studies have shown that α-syn preformed fibrils (α-syn PFF) act as seeds that promote misfolding and aggregation of endogenous α-syn in neighboring neurons, consistent with a prion-like mode of propagation [9,10,11]. Importantly, vagotomy has been reported to reduce the spread of α-syn pathology and is associated with attenuation of dopaminergic neuronal loss and motor deficits [12].

To further validate the Braak hypothesis, gastrointestinal α-syn PFF injection models have been developed that recapitulate key features of PD and enable monitoring of pathological α-syn propagation. These models have become important tools for investigating gut-originating α-syn transmission. However, substantial methodological heterogeneity exists among studies with respect to animal species and age, injection sites, and dosage [12,13,14,15,16]. For example, injection of α-syn PFF into the muscular layers of the pylorus and duodenum of C57BL/6 mice resulted in modest accumulation of phosphorylated α-syn (p-α-syn) in the ventral midbrain at 3 months post-injection, followed by robust p-α-syn accumulation in the ventral midbrain and hippocampus at 7 months, accompanied by significant dopaminergic neuronal loss and motor impairments [12]. In contrast, α-syn PFF injection into the colon of rats or non-human primates led to only mild p-α-syn accumulation in the dorsal motor nucleus of the vagus (DMV) at 1 month, with no detectable p-α-syn pathology in the brain at 12 months [14]. Moreover, age has been identified as a critical determinant of efficient α-syn propagation following α-syn PFF gut seeding, with aging increasing susceptibility to α-syn misfolding [16]. Importantly, most existing models are established on the C57BL/6 background, despite genetic susceptibility being one of the most significant risk factors for PD, particularly in individuals carrying SNCA mutations such as A53T. How genetic background influences the efficiency of gut-to-brain propagation of α-syn pathology remains largely unknown.

In addition, the vagus nerve, representing the most direct anatomical conduit between the gut and the brain, is considered a central pathway for α-syn transmission in the Braak hypothesis. While growing evidence supports the notion that α-syn pathology can spread from the gut to the brain via the vagus nerve, conflicting findings have been reported. Arotcarena et al. demonstrated that injection of purified LBs derived from PD patient brains into the gastric wall and duodenum of olive baboons did not result in detectable α-syn pathology within the vagus nerve, but led to a marked increase in α-syn levels in whole blood [17]. Van Den Berge et al. proposed that α-syn pathology may propagate through the coeliac ganglia in a vagus-independent manner [16]. These discrepancies highlight that whether gut-derived α-syn pathology ascends to the brain via the vagus nerve remains an unresolved question.

Beyond serving as a passive conduit, it is also unclear whether the vagus nerve actively participates in PD pathogenesis by modulating intestinal microbiota. The gut microbiota, a key mediator of gut–brain communication, has been increasingly implicated in PD. Clinical studies consistently report gut dysbiosis in patients with PD, characterized by an enrichment of pro-inflammatory taxa such as Lactobacillaceae and Enterobacteriaceae, along with a reduction in beneficial short-chain fatty acid-producing bacteria, including Ruminococcaceae, Lachnospiraceae, and Prevotellaceae [18,19,20]. Such microbial alterations may contribute to PD pathogenesis by impairing intestinal barrier integrity, triggering local and systemic inflammation, and modulating α-syn conformation and aggregation [21,22,23]. However, in gut-originating α-syn PFF models, how initial pathological α-syn aggregation in the intestine influences microbial ecology and function remains poorly understood. Emerging evidence suggests that neurodegenerative diseases frequently share intersecting metabolic disruptions driven by multimodal brain–body pathways, with metabolic dysfunction serving as a bridge between brain alterations and systemic disease [24,25]. This broader brain–body network perspective provides a useful framework for understanding how vagal alterations may extend beyond α-syn pathology to influence systemic metabolic homeostasis. Recent genomic and cell-type-specific studies have demonstrated that genetic background shapes structural phenotypes through distinct biological pathways and cell-specific vulnerabilities, highlighting the importance of precisely controlled genetic backgrounds in disease modeling [26]. Therefore, to explore how genetic background modulates gut-to-brain α-syn propagation, we included genetically distinct A53T transgenic mice in the present study.

The gastrointestinal α-syn PFF injection model, while promising, requires further investigation to address key questions regarding gut-to-brain α-syn propagation in PD. In the present study, we used this model in C57BL/6J and A53T transgenic mice, combined with truncal vagotomy, to examine gut-to-brain propagation of α-syn pathology. We further assessed associated changes in gut microbiota and their relationship with truncal vagotomy. In addition, we included A53T transgenic mice to examine gut-to-brain α-syn pathology under a different genetic background. By integrating pathological, behavioral, and microbiome analyses, this study provides multi-level observations relevant to gut-originating α-syn pathology and its potential links to PD.

## 2. Materials and Methods

### 2.1. Animals

Male C57BL/6J mice were purchased from Shandong Pengyue Laboratory Animal Technology Co., Ltd. (Jinan, China) and underwent surgical procedures at 2 months of age (body weight approximately 21–25 g). Homozygous A53T α-synuclein (α-syn) transgenic mice of the M83 line (Tg(SNCA)83Vle) were obtained from Shanghai Model Organisms Center, Inc. (Shanghai, China) and underwent surgical procedures at 4 months of age. The genotype of A53T Tg mice was identified by probe-based real-time quantitative PCR. Primer and probe sequences were obtained from The Jackson Laboratory (Table 1). Animals were housed under specific pathogen-free conditions at a controlled temperature of 24 ± 2 °C with a 12 h light/dark cycle and had free access to food and water. Mice were allocated to experimental groups by body weight matching to ensure comparable baseline weights, with surgeries performed in parallel across groups to minimize order effects and cage positions balanced. Only the operator performing injections was aware of group allocation; all outcome assessments and data analyses were performed blinded. All experimental procedures were approved by the Institutional Animal Care and Use Committee at Shanghai Institute of Materia Medica. IACUC No. 2022-10-GMY-32.

### 2.2. Preparation of α-Synuclein Preformed Fibrils (PFF)

Recombinant human monomeric α-syn (Novoprotein, Suzhou, China) was dissolved in phosphate-buffered saline (PBS) at a concentration of 5 mg/mL. The solution was incubated at 37 °C with constant agitation (Eppendorf ThermoMixer C, Hamburg, Germany) at 1000 rpm for 7 days to induce fibril formation, after which it was stored at −80 °C. Before experimental application, PFF were thawed at room temperature and diluted with PBS to a final concentration of 2.5 mg/mL. Sonication was performed using a probe sonicator (Tuohe Electromechanical Technology Co., Ltd., JY88-IIN, Shanghai, China) at 20% amplitude for 60 cycles, with each cycle consisting of 0.5 s on and 0.5 s off, for a total sonication time of 30 s.

### 2.3. Thioflavin T (ThT) Assay

Monomeric α-syn was dissolved in PBS (pH 7.2) at a concentration of 160 ng/μL. A total of 10 μg α-syn was incubated with ThT (25 μM) (Merck, 596200, Darmstadt, Germany) in a 62.5 μL reaction mixture at 37 °C in a temperature-controlled microplate shaker (Hangzhou Allsheng Instruments Co., Ltd., MB100-2A, Hangzhou, China) with continuous shaking at 1000 rpm for 7 days. Fluorescence intensity was measured using a microplate reader (Envision, PerkinElmer, Hopkinton, MA, USA), with excitation and emission wavelengths set at 450 nm and 485 nm, respectively. Data were collected at the following time points: 0 h, 6 h, 24 h, 48 h, 72 h, 96 h, 120 h, 144 h, and 168 h. Fluorescence intensity was positively correlated with the extent of protein aggregation.

### 2.4. Duodenal Muscularis Injection of α-Syn PFF and Truncal Vagotomy

Mice were anesthetized via intraperitoneal injection of a combined anesthetic consisting of 8.3% (*v*/*v*) Zoletil 50 and 2.7% (*v*/*v*) xylazine hydrochloride at a dose of 5 mL/kg. All surgical procedures were performed under a stereomicroscope. α-Syn PFF (2.5 mg/mL) were injected into the duodenal muscularis using a 34-gauge microsyringe (Hamilton, 701RN, Bonaduz, Switzerland). In total, 2.5 μL was injected at each of five sites spaced 0.5 cm apart, resulting in a total dose of 31.25 μg α-syn PFF per mouse. Control mice received equal volumes of PBS at the same injection sites. Following injection, the abdominal muscle layer and skin were sutured separately using 4-0 absorbable surgical sutures (Jinhuan Medical, LJH406, Yuhuan, China). The incision site was disinfected with povidone-iodine, and mice were returned to their cages in a supine position to avoid pressure on the wound. For mice in the truncal vagotomy plus α-syn PFF (TV + PFF) group, α-syn PFF duodenal injection was performed immediately after vagotomy. The esophagus, connected to the stomach, was gently exposed using a glass probe, allowing visualization of the vagus nerve tightly associated with the esophagus. Bilateral vagal trunks were carefully isolated and transected using fine forceps. To ensure complete truncal vagotomy, transection was performed at a site away from the stomach and toward the cervical direction, as vagal branching is more extensive near the stomach. All surgical procedures were performed under combined anesthesia and strictly maintained under sterile conditions. After surgery, all animals received daily postoperative monitoring, including assessment of mental status, food and water intake, wound healing, and general behavior. Mice were sacrificed for tissue collection at 7 days and 4 months after α-syn PFF injection.

### 2.5. Grip Strength Test

Mice were placed on a metal grid (Shanghai Kuoyun Instrument Equipment Co., Ltd., KW-GSM, Shanghai, China) and allowed to grasp it with all four limbs. The tail was gently pulled backward until the mouse released the grid, and the maximal grip force was recorded. Each mouse was tested three times, and the average value was calculated. Grip strength was expressed in gram-force (gf).

### 2.6. Rotarod Test

Mice were placed on an accelerating rotating rod with a diameter of 3 cm (Shanghai Kuoyun Instrument Equipment Co., Ltd., KY-ROTD, Shanghai, China), with one empty lane between adjacent mice to minimize interference. The rotation speed increased from 5 to 40 rpm over a period of 300 s. The latency to fall was recorded, with a maximum cutoff time of 300 s. Each mouse underwent three trials with an inter-trial interval of at least 30 min, and the average latency was used for analysis. Based on previous studies, the cutoff time for the rotarod test was set at 300 s, as this time point effectively distinguishes motor deficits without causing excessive fatigue [27].

### 2.7. CatWalk Gait Test

Gait analysis was performed using the CatWalk™ XT automated gait analysis system (Noldus Information Technology, Wageningen, The Netherlands) under dark conditions. Mice were habituated and trained one day prior to testing. Locomotion was recorded by a high-speed camera. Stride length and swing speed of both forelimbs and hindlimbs were calculated to assess motor coordination.

Given that the core pathological features of Parkinson’s disease include motor deficits and dopaminergic neuron loss, the primary objective of this study was to determine whether intestinal α-syn PFF injection could successfully induce these central pathological features. Therefore, motor behavioral assessments were prioritized at 3 and 4 months post-injection, whereas early non-motor symptoms were not evaluated in the present study and remain to be addressed in future longitudinal investigations.

### 2.8. Tissue Collection and Experimental Applications

Mice were anesthetized by intraperitoneal injection of a combined anesthetic consisting of 8.3% (*v*/*v*) Zoletil 50 and 2.7% (*v*/*v*) xylazine hydrochloride at a dose of 5 mL/kg, followed by transcardial perfusion with ice-cold saline. All tissues were collected on ice.

For mice at 7 days after α-syn PFF injection, the entire intestine was collected and immediately fixed in 4% paraformaldehyde (PFA) at 4 °C. After 24 h, the fixative was replaced with fresh 4% PFA. The duodenal injection segment was then processed for paraffin embedding followed by sectioning and used for hematoxylin and eosin staining and p-α-syn immunofluorescence staining.

For C57BL/6J and A53T Tg mice at 4 months after α-syn PFF injection, brain and intestinal tissues were collected using the same procedures. The whole brain was removed and divided into left and right hemispheres along the midline. The left hemisphere was fixed in 4% PFA and stored at 4 °C, followed by dehydration and serial cryosectioning. The substantia nigra and striatum were used for p-α-syn immunofluorescence staining and immunohistochemistry staining. From the right hemisphere, the striatum was dissected and placed into sterile, nuclease-free tubes and stored at −80 °C for Western blot analysis of p-α-syn protein expression, while the remaining tissue was stored at −80 °C. The intestinal tissue was processed according to the procedure described for sample collection from mice at 7 days post α-syn PFF injection.

### 2.9. Immunofluorescence Staining and Quantitative Analysis

Left brain hemispheres were dehydrated in 30% (*w*/*v*) sucrose, embedded in OCT compound, and sectioned coronally at 20 μm thickness using a frozen sectioning system. Sections were stored in cryoprotectant at 4 °C. Fixed duodenal tissues were dehydrated through graded ethanol, embedded in paraffin, and sectioned transversely at 4 μm. Brain cryosections and deparaffinized duodenal sections were permeabilized with 0.3% Triton X-100 for 10 min, blocked in 10% goat serum for 2 h, and incubated overnight at 4 °C with primary antibodies against pSer129-α-syn (Abcam, ab51253, 1:1000, Cambridge, UK), tyrosine hydroxylase (TH; Merck, MAB318, 1:2000, Darmstadt, Germany). Sections were then incubated with Alexa Fluor 488-conjugated goat anti-rabbit IgG (Invitrogen, A-21206, 1:500, Carlsbad, CA, USA) and Alexa Fluor 555-conjugated goat anti-mouse IgG (Invitrogen, A-31570, 1:500, Carlsbad, CA, USA) for 2 h at room temperature in the dark. Slides were mounted using an antifade mounting medium containing DAPI (Beyotime, P0131, Shanghai, China) and imaged using an Olympus FV3000 confocal microscope. Integrated density was quantified using ImageJ (version 1.54f). For duodenal p-α-syn immunofluorescence staining, the integrated density of p-α-syn in the muscular layer was quantified across four fields per sample, and the average value was calculated to represent the integrated density of p-α-syn in the duodenal muscular layer for each mouse. For p-α-syn immunofluorescence staining in the substantia nigra, the integrated density of p-α-syn was quantified in one to two sections per mouse at the same anatomical level, and the average value was used for statistical analysis. The analyzed sections were selected from a rostrocaudal level corresponding to bregma −3.28 mm. For TH immunofluorescence staining, the number of TH-positive cells was counted across three sections per sample, and the average value was used for statistical analysis. The analyzed sections were selected from rostrocaudal levels corresponding to bregma −3.08 to −3.64 mm.

### 2.10. Immunohistochemistry Staining and Quantitative Analysis

Brain cryosections were treated with 3% hydrogen peroxide to quench endogenous peroxidase activity, permeabilized with 0.3% Triton X-100 for 10 min, and blocked in 10% goat serum for 2 h. Sections were incubated overnight at 4 °C with anti-TH antibody (Merck, MAB318, 1:500, Darmstadt, Germany), followed by incubation with SignalStain Boost IHC Detection Reagent (Cell Signaling Technology, 8125S, Danvers, MA, USA) for 2 h at room temperature. Signal detection was performed using a DAB substrate kit (Cell Signaling Technology, 11724S, 11725S, Danvers, MA, USA). Images were acquired using an inverted microscope. The number of TH-positive neurons in the SN was quantified using ImageJ (1.54f). For each animal, the average value obtained from three coronal sections at comparable anatomical levels was used for statistical analysis. The analyzed sections were selected from rostrocaudal levels corresponding to bregma −3.08 to −3.64 mm.

### 2.11. Hematoxylin and Eosin (HE) Staining

HE staining was performed according to the manufacturer’s instructions (Beyotime, C0105S, Shanghai, China). Deparaffinized duodenal sections were stained with hematoxylin for 4 min, differentiated for 3 s in differentiation solution (0.5% (*v*/*v*) hydrochloric acid in ethanol), rinsed in running water for 10 min, and counterstained with eosin for 7 min. After dehydration, sections were mounted and scanned using the Akoya Vectra Polaris imaging system. Histopathological damage was scored based on villus architecture, crypt number and morphology, epithelial cell injury, and inflammatory cell infiltration, with higher scores indicating more severe tissue damage (Table S1) [28]. For histopathological scoring, three sections per mouse at consistent anatomical locations were evaluated and the average score was used for analysis.

### 2.12. Protein Extraction and Western Blot

All protein extraction procedures were performed on ice. Striatal tissues were placed in grinding tubes containing three stainless steel beads and lysed in RIPA buffer (Epizyme Biotech, PC101, Cambridge, MA, USA) supplemented with 1% protease inhibitor cocktail (Epizyme Biotech, GFR101, Cambridge, MA, USA) and 1% phosphatase inhibitor cocktail (Epizyme Biotech, GFR102, Cambridge, MA, USA). Samples were homogenized using a tissue grinder and centrifuged at 15,000× *g* for 15 min at 4 °C. Supernatants were collected, quantified, mixed with loading buffer, and denatured at 100 °C for 10 min.

Proteins were separated on 10% SDS-PAGE gels (Epizyme Biotech, PG222, Cambridge, MA, USA) and transferred to nitrocellulose membranes. Membranes were blocked in 5% (*w*/*v*) non-fat milk in TBST for 2 h and incubated overnight at 4 °C with antibodies against pSer129-α-syn (Abcam, ab51253, 1:2000, Cambridge, UK) and β-actin (Starter, S0B0005, 1:5000, Wenzhou, China). Membranes were then incubated with HRP-conjugated goat anti-rabbit IgG (ABclonal, AS014, Wuhan, China) for 2 h. Signals were detected using an ECL detection kit (YLESA, 99883-11, Shanghai, China), visualized with e-BLOT software, and optical density was quantified using ImageJ (1.54f).

### 2.13. Metagenomic Sequencing and Analysis

For metagenomic sequencing, mice were selected based on motor performance, with normal motor function in the PBS and TV + PFF groups and motor impairment in the PFF group. Fecal samples were collected from four mice in the PBS group, three mice in the PFF group, and three mice in the TV + PFF group. All samples were immediately stored on dry ice after collection and sent to Shanghai Majorbio Bio-Pharm Technology Co., Ltd. (Shanghai, China) for metagenomic sequencing analysis. DNA was extracted using the FastPure Stool DNA Isolation Kit (Magnetic Bead; MJYH, Shanghai, China). DNA concentration, purity, and integrity were assessed, and samples were fragmented using a Covaris M220 system. Fragments of approximately 350 bp were selected to construct paired-end libraries using the NEXTFLEX^®^ Rapid DNA-Seq Kit (Bioo Scientific, Austin, TX 78744, USA). Sequencing was performed on the Illumina NovaSeq™ X Plus platform. Adapter sequences at both the 3′ and 5′ ends of the reads were trimmed using fastp (version 0.23.0). Reads shorter than 50 bp after trimming or with an average base quality score below 20 were removed, and only high-quality reads were retained for subsequent analyses. Raw sequencing data were processed using the nf-core/taxprofiler pipeline (v1.1.8) for quality control, host read removal, and taxonomic profiling. Functional annotation was performed using HUMAnN 3. Taxonomic profiles generated by Kraken2 and associated metadata were imported into R (v4.4.1) for downstream analyses. Alpha diversity indices, including Chao1, Shannon, and Simpson indices, were calculated to assess within-group diversity. Beta diversity was evaluated using Bray–Curtis distances and visualized by principal coordinates analysis (PCoA). Differences in microbial community structure were assessed using permutational multivariate analysis of variance (PERMANOVA, adonis function in the vegan R package, v4.4.1). Relative abundances of taxa at different taxonomic levels were calculated. Linear discriminant analysis effect size (LEfSe) was used to identify microbial biomarkers based on statistical significance and effect size (LDA score). Functional differences between experimental groups were further analyzed using STAMP to identify significantly enriched or depleted pathways.

### 2.14. Statistical Analysis

Data were analyzed using GraphPad Prism (version 10.1). Normality was assessed using the Shapiro–Wilk test, and homogeneity of variance was evaluated using Levene’s test. Group comparisons were performed using unpaired two-tailed Student’s *t*-test for two groups, or one-way ANOVA with Dunnett’s post hoc test for three or more groups. For behavioral data with repeated measurements across time, two-way repeated-measures ANOVA was applied. Results are presented as means ± SEM. Exact p-values are reported alongside effect sizes, including eta squared for t-tests and partial η^2^ for ANOVA, as well as 95% confidence intervals for mean differences. All tests were two-sided, and *p* < 0.05 was considered statistically significant.

## 3. Results

### 3.1. α-Syn PFF Injection Induced Acute α-Syn Pathology in the Duodenum of C57BL/6J Mice

**Figure 1:**
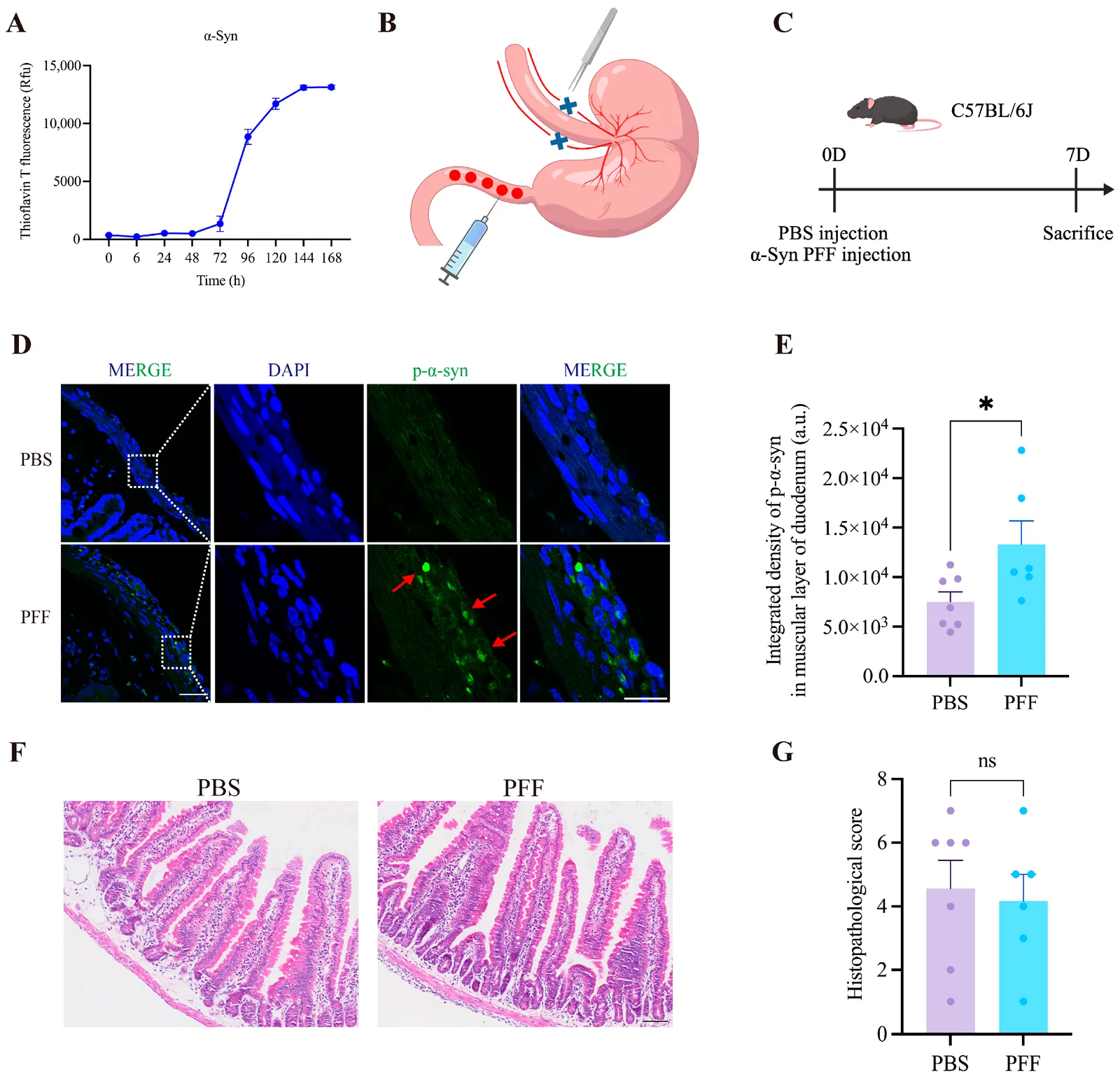
Injection of α-syn PFF into the duodenal muscularis induces acute p-α-syn pathology. (**A**) Thioflavin T (ThT) assay for α-syn aggregation (*n* = 5). α-Syn monomers (160 ng/μL in PBS, pH 7.2) were incubated with ThT (25 μM) at 37 °C for 7 days with continuous shaking (1000 rpm). (**B**) Schematic illustration of α-syn PFF injection at five sites in the duodenal muscular layer and vagotomy. The assembled PFF were diluted to a concentration of 2.5 μg/μL for both sonication and injection. Created with BioGDP.com [29]. (**C**) Experimental timeline showing tissue collection 7 days after injection. (**D**) Representative immunofluorescence staining images of p-α-syn (green) and DAPI (blue) in duodenal tissue. Red arrows indicate p-α-syn deposition. Scale bar: 40 μm and 20 μm. (**E**) Quantification of p-α-syn integrated density in duodenal muscular layer (*n* = 6–7 mice/group). (**F**) Representative HE staining images of duodenal tissue. Scale bar: 100 μm. (**G**) Histopathological scoring of duodenal tissue (*n* = 6–7 mice/group). Data are presented as mean ± SEM. Statistical analysis was performed using Student’s *t*-test. * *p* < 0.05 was considered statistically significant; ns, not significant. PBS group, mice receiving PBS injection into the duodenal muscular layer; PFF group, mice receiving α-syn PFF injection into the duodenal muscular layer.

Thioflavin T (ThT) is a fluorescent dye commonly used to assess protein aggregation, with fluorescence intensity positively correlated with the extent of aggregation. Co-incubation of α-syn monomers with ThT showed that aggregation began at approximately 72 h, reached a plateau at around 144 h, and exhibited no substantial change in fluorescence intensity at 168 h (Figure 1A). These results indicate the aggregation propensity of α-syn. Prior to α-syn PFF injection, 0.1% fast green (Sigma, F7252, Darmstadt, Germany) dissolved in PBS was injected into the duodenal muscular layer of mice to verify accurate delivery to the intestinal muscle layer and to determine a volume that could be administered without leakage. To establish a gut-originating model of Parkinson’s disease, recombinant human α-syn PFF (2.5 mg/mL) were injected into the muscular layer of the duodenum of C57BL/6J mice at five sites, with 2.5 μL administered per site (Figure 1B). To assess early pathological changes, duodenal tissues were collected and analyzed 7 days after injection (Figure 1C). Immunofluorescence staining revealed a marked increase in p-α-syn accumulation within the duodenal muscularis in α-syn PFF-injected mice compared with PBS-treated controls. Quantitative analysis showed that integrated density of p-α-syn increased from 7502 ± 1015 in the PBS group (*n* = 7) to 13,301 ± 2370 in the PFF group (*n* = 6) (*p* = 0.0368, t(11) = 2.375, mean difference = 5799, 95% CI: 425.9 to 11,171, η^2^ = 0.3391), suggesting successful deposition of p-α-syn in the duodenal muscular layer following α-syn PFF injection (Figure 1D,E). HE staining demonstrated no significant differences between the two groups at 7 days post-injection with respect to villus architecture, crypt number and morphology, epithelial integrity, or inflammatory cell infiltration (Figure 1F,G). These findings suggest that, at this early time point, pathological changes are largely restricted to p-α-syn aggregation and have not yet resulted in overt structural damage to the intestinal tissue.

### 3.2. α-Syn PFF Injection Led to Disruption of Duodenal Structure in C57BL/6J Mice

**Figure 2:**
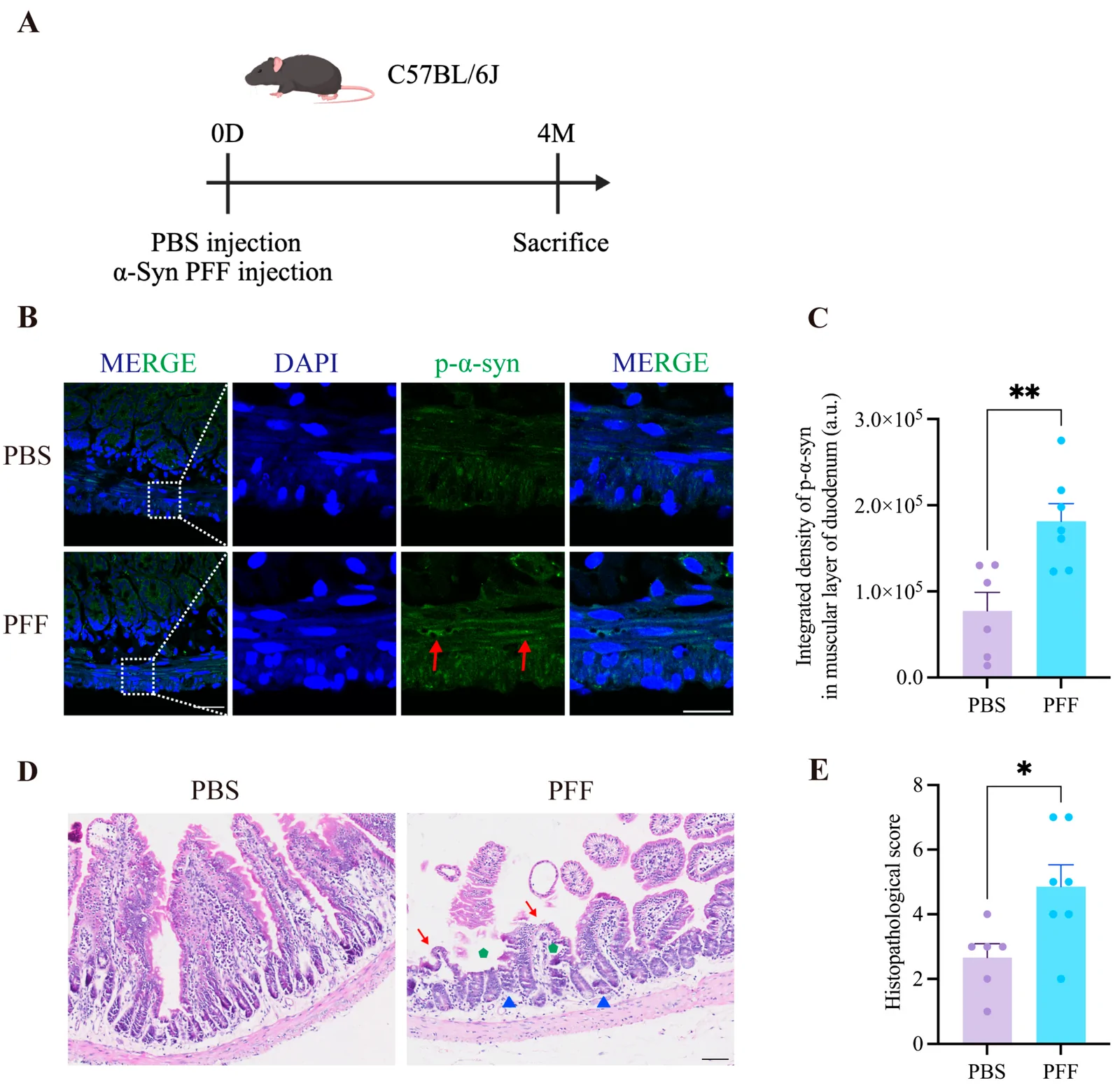
α-Syn PFF injection into the duodenal muscular layer led to intestinal structural disruption at 4 months. (**A**) Experimental timeline showing tissue collection 4 months after injection. (**B**) Representative immunofluorescence staining images of p-α-syn (green) and DAPI (blue) in duodenal tissue. Red arrows indicate p-α-syn deposition. Scale bar: 40 μm and 20 μm. (**C**) Quantification of p-α-syn integrated density in duodenal muscular layer (*n* = 6–7 mice/group). (**D**) Representative HE staining images of duodenal tissue. Red arrows indicate disrupted and markedly shortened villi; blue triangles indicate a reduced number of crypts with a looser arrangement; green pentagons indicate villus vacuolization. Scale bar: 100 μm. (**E**) Histopathological scoring of duodenal tissue (*n* = 6–7 mice/group). Data are presented as mean ± SEM. Statistical analysis was performed using Student’s *t*-test. * *p* < 0.05, ** *p* < 0.01. PBS group, mice receiving PBS injection into the duodenal muscular layer; PFF group, mice receiving α-syn PFF injection into the duodenal muscular layer.

To assess the long-term effects of α-syn PFF injection into the duodenal muscular layer of C57BL/6J mice, p-α-syn pathology in the duodenum was examined at 4 months post-injection (Figure 2A). Consistent with the findings observed at 7 days post-injection, robust p-α-syn deposition persisted in the duodenal muscularis at 4 months. Quantitative immunofluorescence analysis revealed that integrated density of p-α-syn in the duodenal muscular layer increased from 77,373 ± 21,620 in the PBS group (*n* = 6) to 181,441 ± 20,403 in the PFF group (*n* = 7) (*p* = 0.0050, t(11) = 3.495, mean difference = 104,068, 95% CI: 38,533 to 169,604, η^2^ = 0.5262) (Figure 2B,C). Intestinal structural integrity and inflammatory changes were further evaluated by HE staining. Compared with PBS-treated controls, α-syn PFF-injected mice exhibited pronounced disruption of small intestinal architecture, characterized by marked shortening and damage of villi, a reduced number of crypts with a looser arrangement, prominent vacuolization at the villus tips, and a decrease in goblet cell abundance. Histopathological scoring was significantly increased from 2.667 ± 0.422 in the PBS group (*n* = 6) to 4.857 ± 0.670 in the PFF group (*n* = 7) (*p* = 0.0224, t(11) = 2.655, mean difference = 2.190, 95% CI: 0.3746 to 4.006, η^2^ = 0.3906), indicating that duodenal muscularis injection of α-syn PFF disrupts normal intestinal architecture at 4 months post-injection (Figure 2D,E).

### 3.3. p-α-Syn Pathology Ascends from the Gut to the Brain in a Vagus-Related Manner and Induces PD-like Phenotypes in Mice

**Figure 3:**
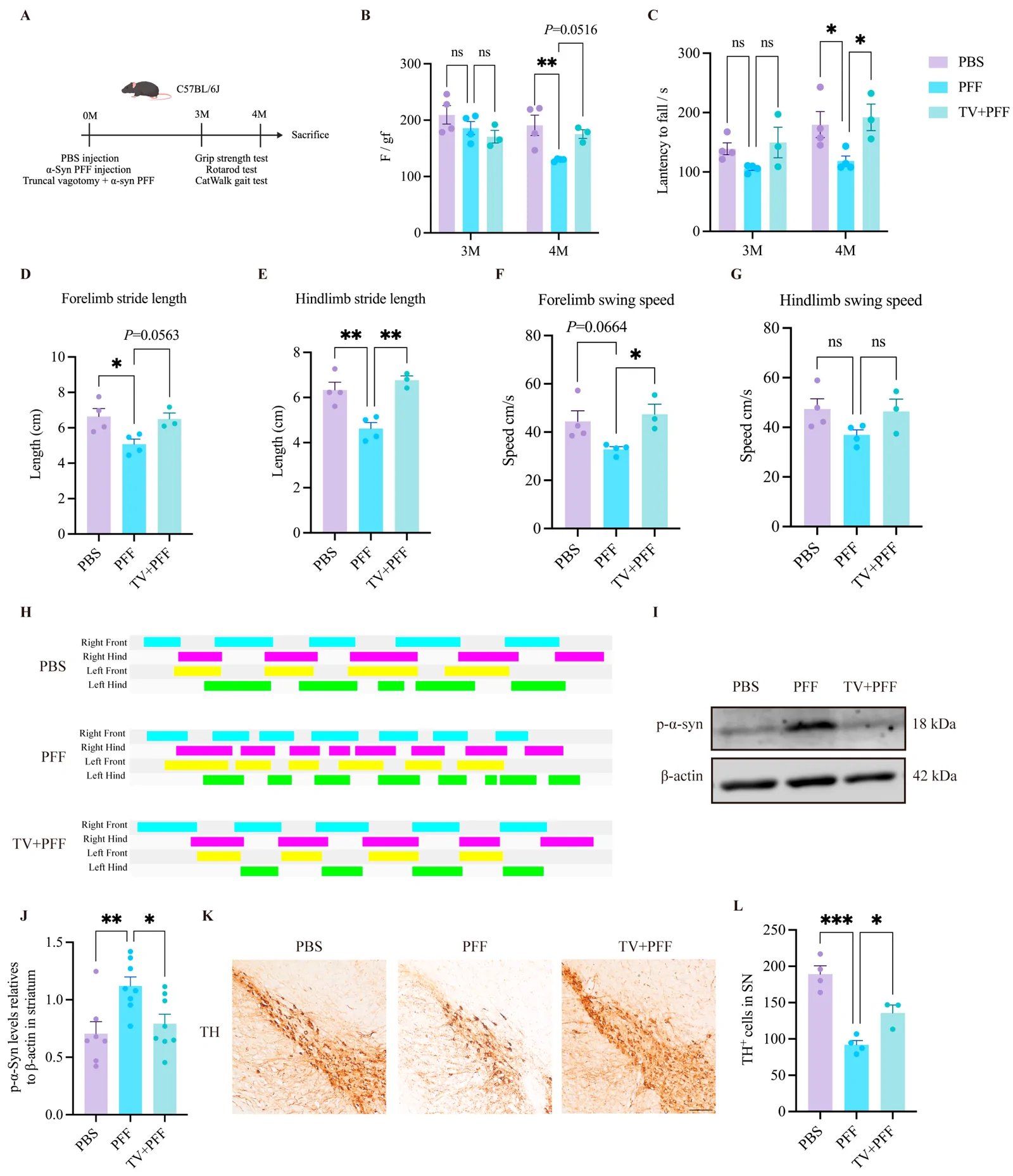
Vagus nerve-related p-α-syn ascension leads to PD-like phenotypes in C57BL/6J mice. (**A**) Behavioral assessments performed at 3 and 4 months after injection. (**B**) Quantification of grip strength performance (*n* = 3–4 mice/group). (**C**) Quantification of rotarod latency to fall (*n* = 3–4 mice/group). (**D**–**G**) Quantification of forelimb and hindlimb stride length and swing speed at 4 months after injection (*n* = 3–4 mice/group). (**H**) Representative footprint patterns from gait analysis at 4 months after injection. Bar length represents ground contact time (s). (**I**) Western blot of p-α-syn expression in striatum. (**J**) Quantification of p-α-syn/β-actin in striatum (*n* = 7–8 mice/group). (**K**) Representative immunohistochemical staining images of tyrosine hydroxylase (TH) positive neurons in substantia nigra (SN). Scale bar: 100 μm. (**L**) Quantification of TH-positive neurons in SN (*n* = 3–4 mice/group). Data are presented as mean ± SEM. Statistical analysis was performed using one-way or two-way ANOVA followed by Dunnett’s test. * *p* < 0.05, ** *p* < 0.01, *** *p* < 0.001. PBS group, mice receiving PBS injection into the duodenal muscular layer; PFF group, mice receiving α-syn PFF injection into the duodenal muscular layer; TV + PFF group, mice subjected to truncal vagotomy followed immediately by α-syn PFF injection.

To determine whether injection of α-syn PFF into the duodenal muscular layer of C57BL/6J mice induces central pathological features of PD, motor behavioral assessments were performed at 3 and 4 months after α-syn PFF injection. Mice were divided into three experimental groups: a PBS-injected control group, an α-syn PFF-injected group, and a group subjected to truncal vagotomy followed immediately by α-syn PFF injection (TV + PFF) (Figure 3A). At 3 months after α-syn PFF injection, no significant reductions were observed in grip strength or in the latency to fall in the rotarod test in mice, indicating that no overt motor deficits were observed (Figure 3B,C). However, at 4 months, mice in the PFF group began to exhibit significant motor impairments. Compared with the PBS group, α-syn PFF-injected mice showed a marked reduction in grip strength and rotarod performance. Grip strength decreased from 190.8 ± 17.92 gf (*n* = 4) to 129.6 ± 1.24 gf (*n* = 4) (*p* = 0.0058, mean difference = −61.25, 95% CI: −103.9 to −18.61), and rotarod latency to fall decreased from 179.7 ± 21.85 s (*n* = 4) to 118.5 ± 8.42 s (*n* = 4) (*p* = 0.0218, mean difference = −61.21, 95% CI: −113.5 to −8.954), indicating impaired motor function. In contrast, the TV+PFF group showed an increase in grip strength and a significant improvement of rotarod latency to fall, with grip strength increasing to 175.3 ± 7.82 gf (*n* = 3) (*p* = 0.0516, mean difference = −45.75, 95% CI: −91.81 to 0.3058) and rotarod latency to fall increasing to 192.0 ± 22.26 s (*n* = 3) (*p* = 0.0113, mean difference = −73.51, 95% CI: −130.0 to −17.07) (Figure 3B,C). Gait analysis further revealed pronounced locomotor deficits in α-syn PFF-injected mice. Compared with PBS controls, the PFF group displayed significantly reduced forelimb and hindlimb stride length. Forelimb stride length decreased from 6.638 ± 0.450 cm (*n* = 4) to 5.079 ± 0.286 cm (*n* = 4) (*p* = 0.0274, mean difference = −1.559, 95% CI: −2.916 to −0.2027), and hindlimb stride length decreased from 6.335 ± 0.344 cm (*n* = 4) to 4.625 ± 0.266 cm (*n* = 4) (*p* = 0.0046, mean difference = −1.710, 95% CI: −2.765 to −0.6550). Forelimb swing speed showed a decreasing trend in the PFF group compared with the PBS group, declining from 44.40 ± 4.337 cm/s (*n* = 4) to 32.86 ± 1.121 cm/s (*n* = 4) (*p* = 0.0664, mean difference = −11.54, 95% CI: −23.95 to 0.8676). By contrast, truncal vagotomy was associated with improved gait parameters. Hindlimb stride length and forelimb swing speed were significantly increased in the TV + PFF group, with hindlimb stride length increasing to 6.767 ± 0.186 cm (*n* = 3) (*p* = 0.0019, mean difference = −2.142, 95% CI: −3.282 to −1.003) and forelimb swing speed increasing to 47.44 ± 4.129 cm/s (*n* = 3) (*p* = 0.0350, mean difference = −14.59, 95% CI: −27.99 to −1.181). Forelimb stride length showed an increasing trend, reaching 6.501 ± 0.338 cm (*n* = 3) (*p* = 0.0563, mean difference = −1.422, 95% CI: −2.888 to 0.04308). No significant differences in hindlimb swing speed were observed among the three groups (Figure 3D–G). Consistently, footprint pattern analysis demonstrated smaller and disorganized footprints in the PFF group relative to PBS controls, whereas the TV + PFF group exhibited larger and more regular footprints, indicating that truncal vagotomy was associated with attenuation of gait disturbances induced by duodenal α-syn PFF injection (Figure 3H).

To further assess whether gut-originating p-α-syn pathology propagates to the brain in this model, p-α-syn pathology in the striatum was examined. Western blot analysis revealed a significant increase in striatal p-α-syn levels in the PFF group compared with PBS controls, with p-α-syn levels relative to β-actin increasing from 0.705 ± 0.104 (*n* = 7) to 1.121 ± 0.077 (*n* = 8) (*p* = 0.0066, mean difference = 0.4158, 95% CI: 0.1169 to 0.7146). This increase was markedly attenuated in the TV + PFF group, in which p-α-syn levels relative to β-actin decreased to 0.792 ± 0.083 (*n* = 8) (*p* = 0.0249, mean difference = 0.3291, 95% CI: 0.04038 to 0.6178) (Figure 3I,J), suggesting that truncal vagotomy was associated with attenuation of gut-to-brain propagation of p-α-syn pathology. Immunohistochemical staining for TH further demonstrated a significant loss of dopaminergic neurons in the SN of α-syn PFF-injected mice. The number of TH-positive neurons decreased from 189.3 ± 11.3 in the PBS group (*n* = 4) to 91.92 ± 5.866 in the PFF group (*n* = 4) (*p* = 0.0001, mean difference = −97.42, 95% CI: −132.1 to −62.74). Importantly, truncal vagotomy was associated with the preservation of dopaminergic neurons, with TH-positive neuron counts of 135.4 ± 11.06 in the TV + PFF group (*n* = 3) (*p* = 0.0260, mean difference = −43.54, 95% CI: −80.99 to −6.070). (Figure 3K,L).

Collectively, these results suggest that injection of α-syn PFF into the duodenal muscular layer of C57BL/6J mice leads to vagus nerve-related ascending propagation of p-α-syn pathology from the gut to the brain within 4 months, resulting in PD-like motor deficits and dopaminergic neurodegeneration.

### 3.4. Injection of α-Syn PFF into the Duodenal Muscularis of C57BL/6J Mice Induces Gut Microbiota Dysbiosis

The preceding results indicate that injection of α-syn PFF into the duodenal muscular layer of C57BL/6J mice induces PD-like pathology in both intestinal and brain tissues. Increasing evidence indicates that the gut microbiota and the central nervous system are connected through a complex bidirectional regulatory network, providing novel insights into the pathophysiological mechanisms of neurodegenerative diseases [30].

To further investigate the impact of duodenal muscularis α-syn PFF injection on gut microbiota structure and function, fecal samples were collected from C57BL/6J mice 4 months after α-syn PFF injection with or without truncal vagotomy and subjected to metagenomic sequencing analysis. Mice were selected for sequencing based on their motor performance at 4 months post-injection, with normal motor function in the PBS group (*n* = 4) and TV + PFF group (*n* = 3) and motor impairment in the PFF group (*n* = 3). This selection strategy was adopted to enhance the biological contrast between groups, allowing us to explore whether gut microbiota composition is associated with the presence or absence of functional deficits. It should be noted that the selection of mice based on motor performance may introduce a potential bias, as microbial composition is sensitive to behavioral and physiological changes accompanying motor impairment. This limitation should be taken into account when interpreting the following microbiome comparisons. Given the exploratory nature of the microbiome analyses, these findings should be interpreted as hypothesis-generating rather than confirmatory. Alpha diversity analysis revealed that the Chao1 index was significantly higher in the PBS group than in the TV + PFF group, whereas no significant differences were observed among the three groups in the Shannon or Simpson indices. Given that the chao1 index is more sensitive to rare taxa, these results suggest substantial differences in species richness between the PBS and TV + PFF groups, while dominant species distribution and evenness remained relatively similar (Figure S1A–C). In the beta diversity analysis, principal coordinates analysis (PCoA) showed differences in gut microbial community structure among the three groups (Figure S1D).

**Figure 4:**
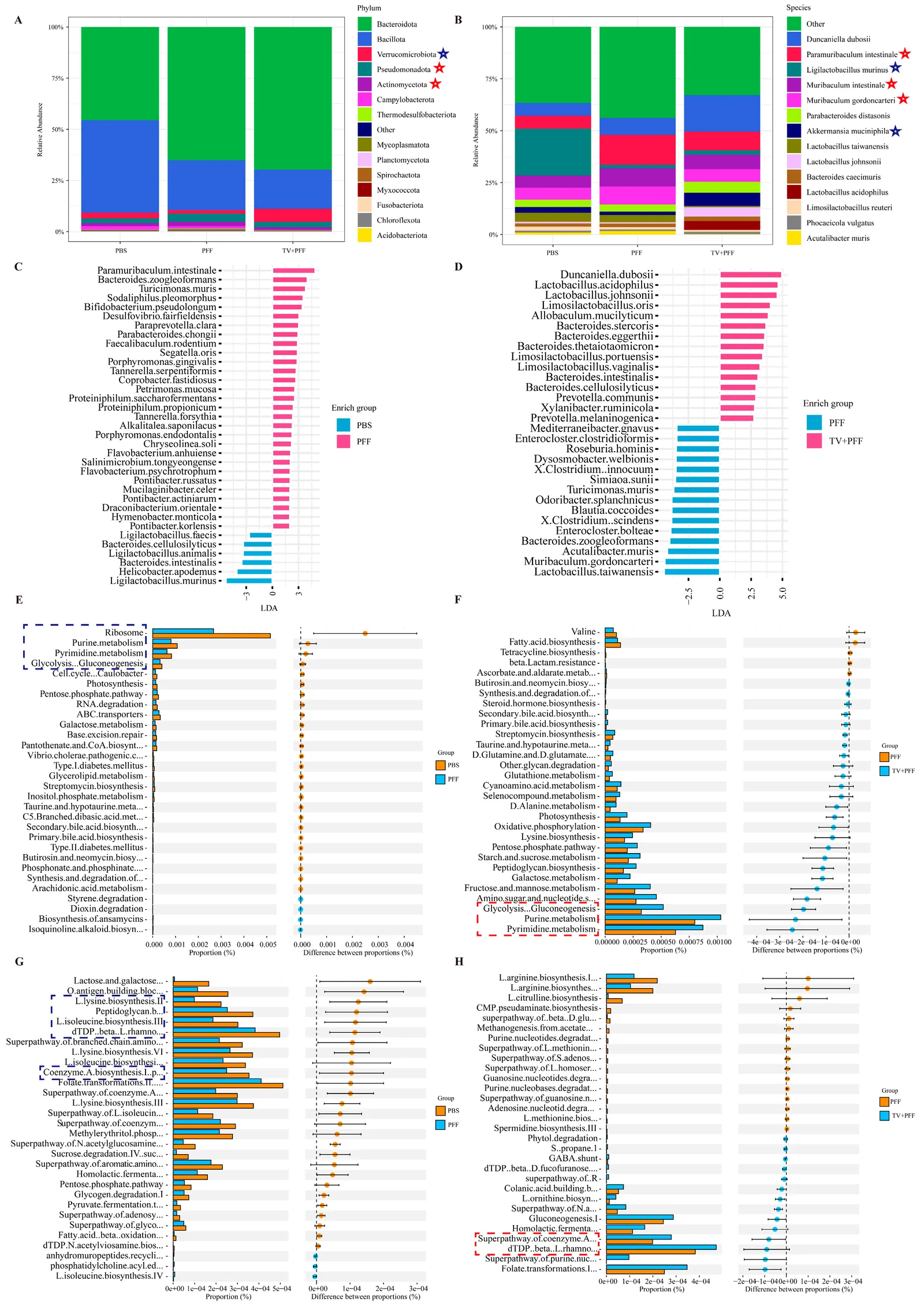
Duodenal muscularis injection of α-syn PFF alters gut microbiota composition and function in C57BL/6J mice. Fecal samples were collected from C57BL/6J mice 4 months after α-syn PFF injection, with or without truncal vagotomy, and subjected to metagenomic sequencing analysis (*n* = 3–4). (**A**,**B**) Phylum-level (**A**) and Species-level (**B**) composition showing shifts in major bacterial taxa across groups. Red stars indicate taxa with increased relative abundance in the PFF group, whereas blue stars indicate taxa with decreased relative abundance. (**C**,**D**) LEfSe analysis identifying differentially abundant species between the PBS and PFF groups (**C**) and between the PFF and TV + PFF groups (**D**). (**E**) KEGG pathway enrichment analysis comparing the PBS and PFF groups, showing broad alterations in core metabolic pathways in the PFF group, predominantly characterized by downregulation. Pathways highlighted in blue boxes are downregulated in the PFF group. (**F**) KEGG pathway enrichment analysis comparing the PFF and TV + PFF groups, showing further modulation of metabolic pathways following truncal vagotomy. Pathways highlighted in red boxes are upregulated in the TV + PFF group. (**G**) MetaCyc pathway enrichment analysis comparing the PBS and PFF groups, showing broad alterations in biosynthetic pathways in the PFF group. Pathways highlighted in blue boxes are downregulated in the PFF group. (**H**) MetaCyc pathway enrichment analysis comparing the PFF and TV + PFF groups, showing alterations in biosynthetic pathways following truncal vagotomy. Pathways highlighted in red boxes are upregulated in the TV + PFF group. PBS group, mice receiving PBS injection into the duodenal muscular layer; PFF group, mice receiving α-syn PFF injection into the duodenal muscular layer; TV + PFF group, mice subjected to bilateral gastric truncal vagotomy followed immediately by α-syn PFF injection.

Further analysis of gut microbiota composition revealed pronounced dysbiosis at the phylum level in the PFF group, exhibiting patterns consistent with those reported in patients with PD [31]. Specifically, the relative abundances of *Pseudomonadota* (formerly *Proteobacteria*) and *Actinomycetota* (formerly *Actinobacteria*) were increased in α-syn PFF-treated mice. Notably, the abundance of *Verrucomicrobiota* (formerly *Verrucomicrobia*) was reduced in the PFF group. These phylum level changes are generally associated with a shift toward a pro-inflammatory microbial profile and disruption of intestinal microbial homeostasis [32,33]. Truncal vagotomy was associated with shifts in the abundance of these three phyla but simultaneously exacerbated the enrichment of *Bacteroidota* (formerly *Bacteroidetes*) and the reduction in *Bacillota* (formerly *Firmicutes*) and *Campylobacterota*, suggesting that truncal vagotomy was associated with complex alterations of the gut microbial community rather than a simple return to the PBS control profile (Figure 4A).

At the species level, taxa enriched in the PFF group included *Paramuribaculum intestinale*, *Muribaculum intestinale*, and *Muribaculum gordoncarteri*, among others. Although these taxa have been reported to exert metabolic or barrier-protective functions [23,34,35], their enrichment in this context may represent a compensatory response to intestinal dysfunction rather than a net beneficial effect. *Paramuribaculum intestinale* exerts metabolic regulatory and barrier-protective effects, effectively ameliorating disease phenotypes in mice with metabolism-associated fatty liver disease (MAFLD), and synergistically enhancing the hepatoprotective effects of bupleuri radix polysaccharides in MAFLD mice [34]. *Muribaculum intestinale* has been reported to convert succinate into propionate and to inhibit the colonization of Salmonella typhimurium, and is implicated in the maintenance of intestinal barrier function and colonization resistance [23]. *Muribaculum gordoncarteri* produces uric acid through its metabolic activity, which reduces the infiltration of myeloid-derived suppressor cells and activates CD8^+^ T cells, thereby promoting the transition of an immunosuppressive tumor microenvironment toward an immunologically active state [35]. Concurrently, the relative abundances of *Akkermansia muciniphila* and several *Lactobacillus* species, including *Ligilactobacillus murinus* and *Limosilactobacillus reuteri*, were reduced in the PFF group (Figure 4B). *Akkermansia muciniphila* is a mucin-degrading commensal bacterium with unique physiological properties and exhibits potent anti-inflammatory and metabolic regulatory functions through the expression of specific functional proteins such as Amuc_1100, thereby directly contributing to the maintenance of mucosal homeostasis [36]. *Lactobacillus* represent an important group of lactic acid-producing bacteria in the gut and have been widely demonstrated to exert immunomodulatory and anti-inflammatory effects. *Ligilactobacillus murinus* alleviates crotonis fructus-induced intestinal toxicity and acts synergistically with processed crotonis fructus cream to more effectively treat ulcerative colitis [37]. *Limosilactobacillus reuteri*, a widely used probiotic, maintains intestinal barrier integrity and immune homeostasis and regulates both local and systemic immune responses under homeostatic and pathological conditions [38]. The reduction in these beneficial bacteria may therefore directly compromise intestinal immune regulation and impair barrier function.

The gut microbiota alterations associated with truncal vagotomy were selective. Truncal vagotomy was associated with attenuated the enrichment of certain PFF-associated taxa, such as *Paramuribaculum intestinale* and *Muribaculum intestinale*, and with increased the relative abundances of selected beneficial bacteria including *Akkermansia muciniphila* and *Ligilactobacillus murinus*. Conversely, truncal vagotomy was also associated with exacerbated enrichment of some commensal taxa, such as *Duncaniella dubosii*, and with further reduced abundance of lactic acid-producing beneficial bacteria including *Lactobacillus taiwanensis* (Figure 4B). However, the absence of a vagotomy-only control group precludes determination of whether these selective changes are attributable to the loss of vagal signaling, the interruption of α-syn pathology propagation, or a combination of both.

To further compare differences in gut microbiota composition among groups, LEfSe analysis was performed. Compared with the PBS group, the PFF group exhibited a significant increase in the abundance of 29 bacterial species and a significant decrease in 6 species (Figure 4C). In contrast, when compared with the PFF group, the TV + PFF group showed significant enrichment of 31 species and significant reduction of 127 species; the top 15 species by LDA score were selected for visualization (Figure 4D). Notably, truncal vagotomy was associated with increased relative abundances of *Bacteroides cellulosilyticus* and *Bacteroides intestinalis*, and decreased relative abundances of *Bacteroides zoogleoformans*, *Desulfovibrio fairfieldensis*, *Mucilaginibacter celer*, *Parabacteroides chongii*, *Proteiniphilum propionicum*, and *Turicimonas muris*. Importantly, the magnitude of gut microbiota alteration associated with truncal vagotomy exceeded that observed following α-syn PFF injection alone.

To explore potential functional differences in gut microbiota among groups, HUMAnN-predicted KEGG and MetaCyc pathways were statistically analyzed using STAMP. KEGG analysis revealed that core metabolic pathways, including ribosome biogenesis, purine and pyrimidine metabolism, and glycolysis/gluconeogenesis, were downregulated in the PFF group compared with the PBS group, suggesting that α-syn PFF-induced intestinal pathology suppresses fundamental microbial metabolic activity. Following truncal vagotomy, purine and pyrimidine metabolism, and glycolysis/gluconeogenesis pathways were upregulated, suggesting an increase in microbial energy metabolism and biosynthetic capacity (Figure 4E,F). MetaCyc pathway enrichment analysis further revealed functional changes related to amino acid biosynthesis and cell wall synthesis. In the PFF group, biosynthetic pathways for L-isoleucine, L-lysine, dTDP-β-L-rhamnose, coenzyme A, and peptidoglycan were broadly downregulated (Figure 4G,H). Notably, downregulation of branched-chain amino acid (BCAA) biosynthesis pathways warrants particular attention, as plasma BCAA levels are significantly reduced in patients with PD and decline with increasing disease severity. In humans, BCAAs are primarily derived from dietary sources, and the gut microbiota plays a critical role in dietary nutrient metabolism; thus, alterations in plasma BCAA levels in PD patients may be closely linked to gut microbiota dysregulation [39]. In addition, downregulation of dTDP-β-L-rhamnose and peptidoglycan biosynthesis pathways suggests that α-syn PFF may suppress bacterial cell wall synthesis, thereby impairing microbial colonization capacity. Following truncal vagotomy, upregulation of these pathways was observed, particularly in dTDP-β-L-rhamnose and coenzyme A biosynthesis pathways (Figure 4G,H). Coenzyme A is a central cofactor in energy metabolism and fatty acid synthesis, and restoration of its biosynthetic pathway may reflect an overall improvement in microbial metabolic activity.

Overall, duodenal muscularis injection of α-syn PFF not only induces intestinal p-α-syn pathology but also disrupts the gut microenvironment, leading to concurrent structural and functional dysbiosis of the gut microbiota. Although certain taxa with reported protective functions were enriched, this likely reflects an incomplete compensatory response, as evidenced by the depletion of beneficial bacteria and widespread suppression of microbial metabolic pathways. In contrast, truncal vagotomy was associated with alterations in gut microbiota structure and function. However, because a vagotomy-only control group was not included in this study, we cannot determine whether the observed microbiota alterations are attributable to the physiological consequences of surgical denervation alone, the interruption of α-syn pathology propagation, or an interaction between these factors. Therefore, although these data suggest an association between vagal integrity and gut microbiota composition in the context of α-syn pathology, causal conclusions about the role of the vagus nerve in microbiota regulation are not warranted without additional studies incorporating appropriate control groups.

### 3.5. Vagus-Related Ascending p-α-Syn Propagation and PD-like Phenotypes in A53T Transgenic Mice

Notably, in the wild-type C57BL/6J mouse model described above, although p-α-syn pathology propagated to the striatum and was accompanied by motor impairment, no prominent p-α-syn aggregates were detected in the SN (Figure S2A–D). This may be related to the relatively low expression of α-syn in C57BL/6J mice, which could limit further amplification and aggregation of pathological α-syn [40]. To further examine gut-to-brain propagation of α-syn pathology in a different experimental context, we next investigated this process in transgenic mice expressing human A53T mutant α-synuclein (A53T Tg mice), which are maintained on a B6C3 genetic background and exhibit increased aggregation propensity [41].

**Figure 5:**
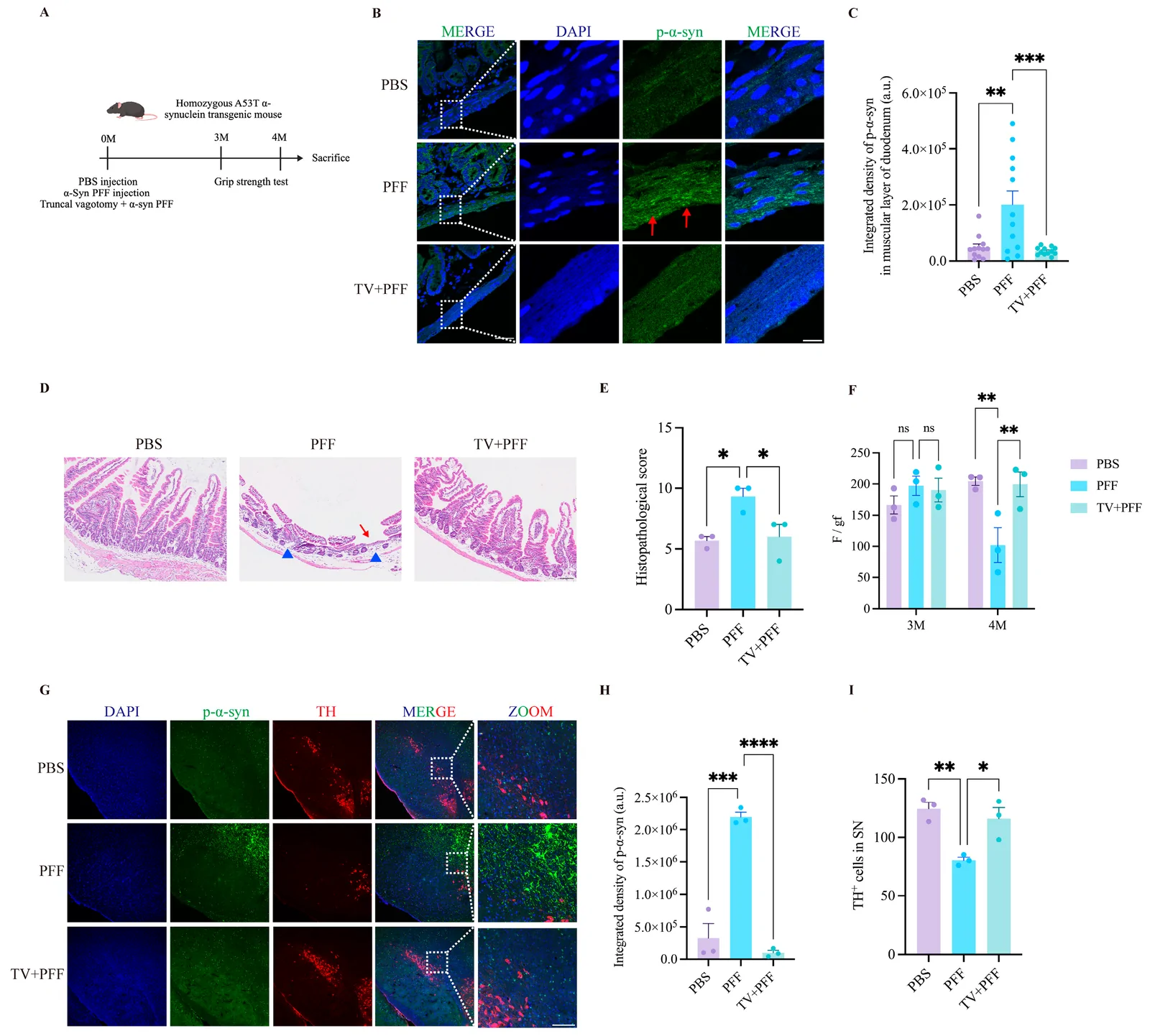
Vagus nerve-related p-α-syn ascension leads to PD-like phenotypes in A53T transgenic mice. (**A**) Experimental timeline showing tissue collection 4 months after injection. (**B**) Representative immunofluorescence staining images of p-α-syn (green) and DAPI (blue) in duodenal tissue. Red arrows indicate p-α-syn deposition. Scale bar: 40 μm and 20 μm. (**C**) Quantification of p-α-syn integrated density in duodenal muscular layer (*n* = 3 mice/group). (**D**) Representative HE staining images of duodenal tissue. Red arrows indicate disrupted and markedly shortened villi; blue triangles indicate a reduced number of crypts with a looser arrangement. Scale bar: 100 μm. (**E**) Histopathological scoring of duodenal tissue (*n* = 3 mice/group). (**F**) Quantification of grip strength performance (*n* = 3 mice/group). (**G**) Representative immunofluorescence staining images of p-α-syn (green), tyrosine hydroxylase (TH; red), and DAPI (blue) in SN. Scale bars: 100 μm and 30 μm. (**H**) Quantification of p-α-syn integrated density in the brain (*n* = 3 mice/group). (**I**) Quantification of TH-positive neurons in SN (*n* = 3 mice/group). Data are presented as mean ± SEM. Statistical analysis was performed using one-way or two-way ANOVA followed by Dunnett’s test. * *p* < 0.05, ** *p* < 0.01, *** *p* < 0.001, **** *p* < 0.0001. PBS group, mice receiving PBS injection into the duodenal muscular layer; PFF group, mice receiving α-syn PFF injection into the duodenal muscular layer; TV + PFF group, mice subjected to bilateral gastric truncal vagotomy followed immediately by α-syn PFF injection.

Homozygous A53T Tg mice received the same dose of α-syn PFF into the duodenal muscularis as administered to C57BL/6J mice, and motor behavioral assessments were conducted at 3 and 4 months post-injection (Figure 5A). p-α-Syn accumulation was observed in the duodenal muscular layer of homozygous A53T Tg mice at 4 months post-injection. Quantitative immunofluorescence analysis showed that the integrated density of p-α-syn in the intestinal muscularis increased from 47,913 ± 12,249 in the PBS group (*n* = 3) to 200,580 ± 50,050 in the PFF group (*n* = 3) (*p* = 0.0019, mean difference = 152,668, 95% CI: 55,169 to 250,166). In contrast, intestinal p-α-syn levels were significantly reduced in the TV + PFF group, with integrated density of p-α-syn decreasing to 35,378 ± 3967 (*n* = 3) (*p* = 0.0008, mean difference = 165,203, 95% CI: 67,704 to 262,701) (Figure 5B,C). Interestingly, HE staining revealed substantially more severe intestinal pathology in A53T Tg mice. In the PFF group, small intestinal villi were severely atrophic and flattened, with intact villi being nearly absent. Goblet cells and crypts were markedly reduced, and overall tissue architecture was profoundly disrupted. Correspondingly, histopathological scores increased from 5.67 ± 0.33 in the PBS group (*n* = 3) to 9.33 ± 0.67 in the PFF group (*n* = 3) (*p* = 0.0201, mean difference = 3.667, 95% CI: 0.7511 to 6.582). In contrast, mice subjected to truncal vagotomy showed relatively intact intestinal architecture, with more densely arranged villi and partially restored goblet cell and crypt numbers. Although mild vacuolization at the villus tips persisted, histopathological scores were significantly reduced to 6.00 ± 1.00 in the TV + PFF group (*n* = 3) (*p* = 0.0298, mean difference = 3.333, 95% CI: 0.4177 to 6.249) (Figure 5D,E).

Behaviorally, no significant reduction in grip strength was detected in A53T Tg mice at 3 months post-injection. However, compared with the PBS group, the PFF group exhibited a significant decline in grip strength at 4 months post-injection, with grip strength decreasing from 204.4 ± 6.56 gf (*n* = 3) to 101.9 ± 27.71 gf (*n* = 3) (*p* = 0.0035, mean difference = −102.6, 95% CI: −167.4 to −37.76). Grip strength was significantly higher in the TV + PFF group compared with the PFF group, with the grip strength rising to 199.8 ± 20.00 gf (*n* = 3) (*p* = 0.0049, mean difference = −97.89, 95% CI: −162.7 to −33.09) (Figure 5F).

In the central nervous system, extensive p-α-syn accumulation was observed in the substantia nigra and surrounding brain regions of α-syn PFF-injected A53T Tg mice. Quantitative analysis showed that integrated density of p-α-syn increased substantially from 330,968 ± 218,604 in the PBS group (*n* = 3) to 2,195,247 ± 72,209 in the PFF group (*n* = 3) (*p* = 0.0001, mean difference = 1,864,279, 95% CI: 1,319,358 to 2,409,199). This pronounced accumulation was reduced in mice subjected to truncal vagotomy, with integrated density of p-α-syn decreasing to 99,187 ± 36,544 (*n* = 3) (*p* < 0.0001, mean difference = 2,096,059, 95% CI: 1,551,139 to 2,640,980) (Figure 5G,H). In parallel, immunohistochemical analysis revealed a marked loss of dopaminergic neurons in the SN, with the number of TH-positive neurons decreasing from 124.4 ± 5.60 (*n* = 3) to 80.33 ± 2.60 (*n* = 3) (*p* = 0.0057, mean difference = −44.08, 95% CI: −70.72 to −17.45). In mice subjected to truncal vagotomy, TH-positive neuron counts were higher, reaching 115.9 ± 9.58 (*n* = 3) (*p* = 0.0155, mean difference = −35.58, 95% CI: −62.22 to −8.947) (Figure 5G,I).

Collectively, these findings show that gut-derived α-syn pathology can propagate to the brain and is accompanied by dopaminergic neurodegeneration in both models. The genetic mismatch between B6C3 A53T Tg mice and C57BL/6J wild-type controls precludes attributing severe pathology solely to the transgene. Baseline strain differences may contribute to these phenotypes, a key limitation for cross-model comparisons.

## 4. Discussion

In this study, we demonstrate that injection of α-syn PFF into the duodenal muscular layer of C57BL/6J mice induces vagus nerve-related ascending propagation of p-α-syn pathology to the central nervous system. Disruption of the vagal pathway was associated with attenuation of this propagation, indicating that the vagus nerve may serve as a conduit for p-α-syn transmission, thereby providing experimental support for the Braak hypothesis. In parallel, duodenal α-syn PFF injection induced marked gut microbiota alterations, while truncal vagotomy was associated with changes in microbial composition and predicted metabolic function. In A53T Tg mice, gut-to-brain propagation of p-α-syn pathology and accompanying neurodegenerative changes were also observed following PFF injection. However, given the absence of a genetically matched wild-type control, the extent to which these findings can be attributed to the A53T transgene, as opposed to differences in genetic background, remains unclear. Collectively, our findings suggest an association between gut microbiota alterations, vagus-mediated gut-to-brain α-syn pathology, and neurodegenerative changes in these models, while also indicating a potential influence of host genetic background.

Although gut-originating α-syn PFF injection models are increasingly used to study PD pathogenesis, this field remains in its early stages. Holmqvist et al. first provided experimental evidence that α-syn pathology can propagate from the gut to the brain via the vagus nerve; however, their study focused on α-syn pathology in the DMV only 6 days after injection and did not examine long-term disease progression [13]. Kim et al. subsequently established a standardized gastric and duodenal α-syn PFF injection model and emphasized long-term outcomes, including motor and cognitive impairments assessed 7 months post-injection, but early disease stages were not systematically investigated [12]. Furthermore, several studies failed to assess motor behavior, thereby overlooking one of the most clinically relevant consequences of α-syn pathology, and investigations into gut microbiota alterations in these models have been extremely limited [15,16]. To address these key questions, the present study systematically examined gut-to-brain propagation of p-α-syn pathology. By injecting α-syn PFF into the duodenal muscular layer of C57BL/6J mice and including a truncal vagotomy group, motor behavior was monitored at 3 and 4 months post-injection, and fecal metagenomic sequencing was performed after the onset of motor impairment.

Despite the observation of striatal p-α-syn accumulation and motor impairment in wild-type C57BL/6J mice, we did not detect prominent p-α-syn aggregates in the SN. We speculate that the relatively low endogenous α-syn expression in C57BL/6J mice may limit further amplification of pathological α-syn. In addition, the 4-month observation window may be insufficient for retrograde transport of pathological α-syn from striatal terminals to SN cell bodies. In the gut injection model established by Kim et al. [12], substantial nigral pathology emerged at 7 months, suggesting that long-range retrograde transport and somatic aggregation require extended timeframes. Regional differences in proteostasis between striatal terminals and SN perikarya, as well as astrocytic clearance efficiency, may also contribute to the absence of somatic aggregates at this time point. Consistent with this notion, Van Den Berge et al. proposed that increased α-syn gene dosage markedly enhances gut-induced PD pathology [42]. Challis et al. reported that, compared with wild-type mice, duodenal injection of α-syn PFF induced more pronounced brain p-α-syn accumulation in Thy1-α-syn overexpressing mice, but the critical role of the vagus nerve in mediating this propagation was not directly evaluated [43]. In A53T Tg mice, we observed extensive p-α-syn pathology, including accumulation in the SN and loss of dopaminergic neurons. However, given the lack of a genetically matched wild-type control, these findings should be interpreted with caution, as the contribution of the A53T transgene cannot be distinguished from that of genetic background differences. A proper control would require mice from the B6C3 background or backcrossing the A53T line to a pure C57BL/6J background to generate congenic mice. These observations are consistent with previous studies suggesting that elevated or mutant α-syn expression may influence the development of α-syn pathology [44,45]. However, the present study does not directly distinguish between endogenous and exogenous α-syn species, and therefore does not allow conclusions regarding seeding processes. Clinically, SNCA multiplications are associated with earlier onset and more rapid disease progression, whereas duplications show a milder course resembling sporadic PD [46,47,48,49,50]. Overall, our findings extend existing observations by demonstrating gut-to-brain propagation of α-syn pathology in both C57BL/6J and A53T transgenic mice under the present experimental conditions, while highlighting the potential influence of genetic background and the need for further studies using controlled genetic models. The differential pathological outcomes between C57BL/6J and A53T Tg mice underscore the importance of genetic background in modulating susceptibility to gut-originated α-syn pathology. This observation resonates with recent studies demonstrating that genetic risk and cell-type-specific expression profiles shape distinct structural phenotypes. Kuang et al. found that cortical thickness differences were associated with immune-related processes involving astrocytes and oligodendrocytes, whereas surface area differences related mainly to inhibitory neurons [26]. Extending this framework to our model, it is plausible that genetic background modulates cell-specific α-syn expression, proteostatic capacity, and inflammatory responses, thereby influencing pathological outcomes. Future studies incorporating transcriptomic and cell-type-resolved analyses across genetically diverse hosts would further clarify how genetic susceptibility and cell-specific vulnerabilities interact to drive distinct trajectories in synucleinopathies.

Beyond serving as a transmission route, our data suggest that the vagus nerve may also be involved in modulating neurodegenerative changes, as reductions in p-α-syn pathology and dopaminergic neurodegeneration were observed in both C57BL/6J and A53T mice following truncal vagotomy. Clinically, vagotomy is rarely performed in modern gastrointestinal surgery, although highly selective vagotomy was historically used to treat refractory peptic ulcers. Epidemiological studies on vagotomy and PD risk remain limited. A large Swedish registry-based cohort study reported no overall association between vagotomy and PD risk, although truncal vagotomy appeared to confer a reduced risk, consistent with earlier findings by Svensson et al. [51,52]. Taken together, these findings indicate that the relationship between vagal integrity and PD pathogenesis is complex and requires further investigation, and the potential relevance of vagal interventions in PD remains to be clarified.

It should be acknowledged that the behavioral assessments in this study were primarily focused on motor function, and early non-motor symptoms, such as gastrointestinal dysmotility or olfactory dysfunction, were not systematically evaluated. In the context of the Braak hypothesis, these autonomic manifestations are considered to precede classical motor decline, and their assessment would therefore provide valuable insights into the early pathogenesis of gut-originating α-syn pathology. However, in the present study, the experimental design prioritized the detection of motor deficits at 4 months post-injection to ensure sufficient pathological burden for functional assessment. Future studies incorporating longitudinal assessments of gastrointestinal transit time and olfactory function across multiple time points will be required to fully characterize the trajectory of non-motor symptoms in this model.

It is also important to consider the potential impact of the cross-species seeding in this study, as the α-syn PFF used were derived from recombinant human monomeric α-syn injected into mice. In this regard, Kim directly compared mouse versus human α-syn PFF in a gut injection model and found that only mouse α-syn PFF induced SN pathology at 3 months post-injection, whereas human α-syn PFF did not, indicating a clear species barrier in seeding efficiency [12]. Consistent with this, other studies have also suggested that mouse α-syn PFF may exhibit higher seeding efficiency than human α-syn PFF in inducing α-syn aggregation and spreading [53,54]. Therefore, the motor deficits observed at 4 months post-injection in the present study should be interpreted in light of this species barrier; the timeline may reflect the kinetics of heterologous seeding rather than the intrinsic dynamics of homologous α-syn propagation. Future studies using mouse-derived α-syn PFF would help clarify whether the observed temporal profile is influenced by the cross-species seeding.

Meanwhile, the metagenomic data provide additional insight into the association between vagus nerve and gut microbiota alterations. Injection of α-syn PFF into the duodenal muscular layer was associated with local intestinal p-α-syn pathology and changes in gut microbiota composition resembling those reported in patients with PD [55]. Consistent with clinical observations, the gut microbiota of patients with PD differs from that of healthy individuals, with increased abundances of Bacteroidetes and Proteobacteria reported to be associated with impaired intestinal barrier function and inflammatory responses, which may influence the central nervous system via the gut–brain axis [56,57].

In the present study, truncal vagotomy was associated with alterations in gut microbiota composition. Before discussing these observations, it is important to consider that mice were selected for metagenomic sequencing based on their motor performance at the endpoint. While this approach was chosen to maximize the biological contrast between groups, thereby increasing the sensitivity of microbiome comparisons to detect differences that track with functional status, it also introduces a potential selection bias, as microbiota composition could be influenced by behavioral and physiological changes associated with motor impairment, including reduced physical activity or altered gastrointestinal motility, rather than directly by α-syn pathology or vagal denervation. However, the specific contribution of vagal denervation versus the attenuation of α-syn pathology to the observed compositional shifts cannot be dissociated in this experimental design. Moreover, we emphasize that the metagenomic analyses presented here are exploratory in nature; they were not powered to detect subtle differences in individual taxa or to establish causal relationships between specific microbial features and disease phenotypes. Although the precise role of the vagus nerve in the “gut microbiota–gut–brain” axis remains incompletely understood, emerging evidence suggests its involvement in gut–brain communication. Previous studies have reported that vagal manipulation is associated with changes in gut microbiota composition, inflammation, and behavior [58], and that microbial effects in PD models may be linked to intact vagal signaling [59]. In addition, vagus nerve stimulation has been associated with changes in intestinal and neural function [60,61]. Collectively, these prior findings and our current data are consistent with vagal involvement in the gut microbiota–gut–brain axis. The downregulation of BCAA biosynthesis pathways observed in our metagenomic data is particularly relevant in this context, as BCAA metabolism has been implicated as a key metabolic nexus linking peripheral metabolic states and neurodegeneration [24,62]. Our observation that truncal vagotomy was associated with shifts in microbial metabolic functions raises the possibility that vagal integrity contributes to systemic metabolic regulation beyond its role in α-syn propagation, consistent with the emerging brain–body network view of neurodegenerative pathology. Nevertheless, whether this relationship is causal, and whether the microbial shifts after vagotomy are attributable to surgical sequelae rather than vagal signaling, requires further investigation with appropriate controls. Accordingly, our microbiome findings should be interpreted as hypothesis-generating rather than confirmatory, and they await validation in larger, independently powered cohorts and through functional experiments.

This study has several limitations. First, we did not characterize the specific cell types of p-α-syn-positive cells, which limits the interpretation of the cellular distribution of pathology. Beyond neuronal transmission, glial cells, particularly microglia and astrocytes, may also participate in α-synuclein propagation. Microglia can internalize α-synuclein aggregates and facilitate their intercellular transfer through incomplete degradation and exosome release [63], while astrocytes can take up and transfer α-synuclein via tunneling nanotubes and exosomes [64]. However, glial responses are context-dependent. Microglia can exert either protective effects through phagocytic clearance or detrimental effects through pro-inflammatory cytokine release and oxidative stress, representing a dual role in synucleinopathies [65]. Activated microglia can drive the conversion of astrocytes into a neurotoxic A1 phenotype, forming a self-amplifying inflammatory circuit that exacerbates neurodegeneration [66]. Whether such glial mechanisms are engaged in the vagal-mediated gut-to-brain propagation of α-syn pathology observed in our model remains unknown, and this question awaits investigation in future studies. Second, intestinal p-α-syn deposition was not assessed in C57BL/6J mice subjected to truncal vagotomy followed by PFF injection, which restricts the evaluation of local pathological changes under vagotomy conditions. Additionally, we did not perform quantitative validation of duodenal α-syn pathology using Western blot, which limits quantitative assessment of overall p-α-syn pathology at the duodenal injection site. Third, the lack of a vagotomy-only control group is a limitation of this study. Therefore, we cannot exclude the possibility that the observed changes in the microbiome were influenced by the vagotomy surgery itself. Future studies including this specific control group are needed to dissect the specific contribution of vagal signaling on gut microbiota composition. Fourth, because truncal vagotomy was performed prior to α-syn PFF injection, this study does not allow evaluation of the therapeutic or restorative effects of vagotomy on established pathology. Fifth, truncal vagotomy results in global denervation of the gastrointestinal tract rather than duodenum-specific denervation, which limits our ability to attribute microbiota changes specifically to attenuation of duodenal α-syn pathology propagation. Fecal samples primarily reflect colonic microbiota and may not accurately represent the duodenal microbial compartment, thereby limiting interpretation of region-specific microbial alterations. In addition, as discussed above, the lack of a genetically matched wild-type control for A53T mice constrains the interpretation of genotype-specific effects. Furthermore, we did not compare α-syn PFF derived from different species sources or matched host backgrounds, and the potential influence of species differences between human and mouse α-syn on aggregation and propagation cannot be excluded. Future studies incorporating these controls and analyses will be important to further clarify the mechanisms underlying gut-to-brain α-syn propagation.

In summary, this study examined gut-to-brain propagation of p-α-syn pathology following duodenal muscularis injection of α-syn PFF. The findings suggest that the vagus nerve is involved in this transmission process and is associated with modulation of related pathological and microbiota changes. In addition, gut-to-brain propagation of p-α-syn pathology and neurodegenerative changes were also observed in A53T transgenic mice under the present experimental conditions. By integrating pathological, behavioral, and microbiome analyses, our work provides multi-level observations consistent with the Braak hypothesis. These findings contribute to the characterization of gut-originating α-syn pathology models and may inform future studies aimed at clarifying underlying mechanisms.

## 5. Conclusions

In conclusion, these results align with the Braak hypothesis, showing that gut-derived p-α-syn pathology and associated functional impairments are intimately linked to vagal pathways during their propagation to the brain. Additionally, this gut-origin PD mouse model may serve as a useful tool for future mechanistic investigations.

## Figures and Tables

**Table 1:** Sequences of PCR primers and fluorescent probes for genotyping.

Primer	5′ Label	Sequence 5′ → 3′	3′ Label	Primer Type
oIMR1544	-	CAC GTG GGC TCC AGC ATT	-	Internal positive control forward
oIMR3580	-	TCA CCA GTC ATT TCT GCC TTT G	-	Internal positive control reverse
oIMR7387	-	CTG GAG AAC GCA CTG TAC GC	-	Transgene forward
oIMR7388	-	CCA ATA CGC AGC CCA GTG T	-	Transgene reverse
TmoIMR0107	Fluorophore-1	CCA ATG GTC GGG CAC TGC TCA A	Quencher-1	IC probe
TmoIMR0110	Fluorophore-2	CTG TCC GCC GTG GGC CAC TT	Quencher-2	Tg probe

## Data Availability

All data supporting the findings of this study are included within the manuscript due to the nature of this research.

## Supplementary Materials

The following supporting information can be downloaded at: https://www.mdpi.com/article/10.3390/brainsci16080804/s1, Figure S1: Injection of α-syn PFF into the duodenal muscularis of C57BL/6J mice induces gut microbiota dysbiosis; Figure S2: No detectable signal by immunofluorescence staining or immunohistochemistry staining in the striatum or SN of C57BL/6J mice; Figure S3: The original blots of p-α-syn expression in the striatum of C57BL/6J mice; Table S1: Histopathological scoring criteria for HE-stained duodenal sections.

## Abbreviations

The following abbreviations are used in this manuscript:

PD	Parkinson’s disease
α-syn	α-synuclein
PFF	preformed fibrils
p-α-syn	phosphorylated α-synuclein
LBs	Lewy bodies
Ser129	serine 129
DMV	dorsal motor nucleus of the vagus
Tg(SNCA)83Vle	transgenic mice of the M83 line
ThT	Thioflavin T
gf	gram-force
PFA	paraformaldehyde
HE	hematoxylin and eosin
PCoA	principal coordinates analysis
LEfSe	Linear discriminant analysis effect size
ANOVA	analysis of variance
MAFLD	metabolism-associated fatty liver disease
A53T Tg mice	transgenic mice expressing human A53T mutant α-synuclein
