# Supplementary material for: Duodenal α-Synuclein PFF Injection Induces Vagus-Related Gut-to-Brain Pathology in C57BL/6J and A53T Transgenic Mice
Source: Brain Sci. 2026 Jul 30;16(8):804. doi: 10.3390/brainsci16080804 (PMC13510561; doi:10.3390/brainsci16080804)
Supplement: Supplementary file 1 [file brainsci-16-00804-s001.zip › Supplementary Materials.pdf]

Supplementary Materials

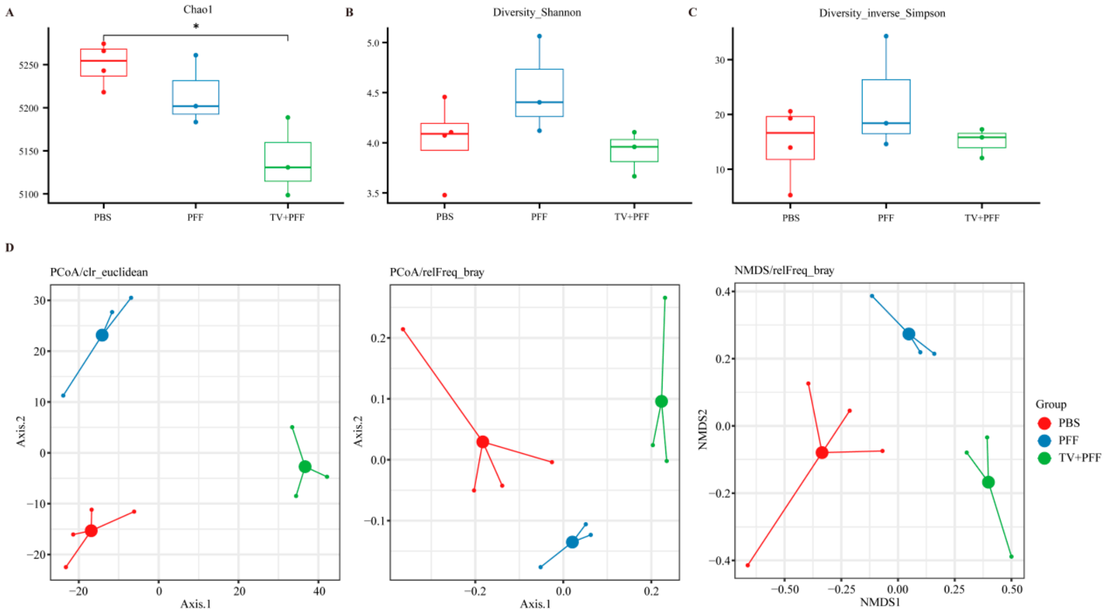

Figure S1. Injection of α-syn PFF into the duodenal muscularis of C57BL/6J mice induces gut microbiota dysbiosis. A. Alpha diversity analysis based on the chao1 index. B. Alpha diversity analysis based on the shannon index. C. Alpha diversity analysis based on the simpson index. D. Beta diversity analysis based on Bray–Curtis distance.

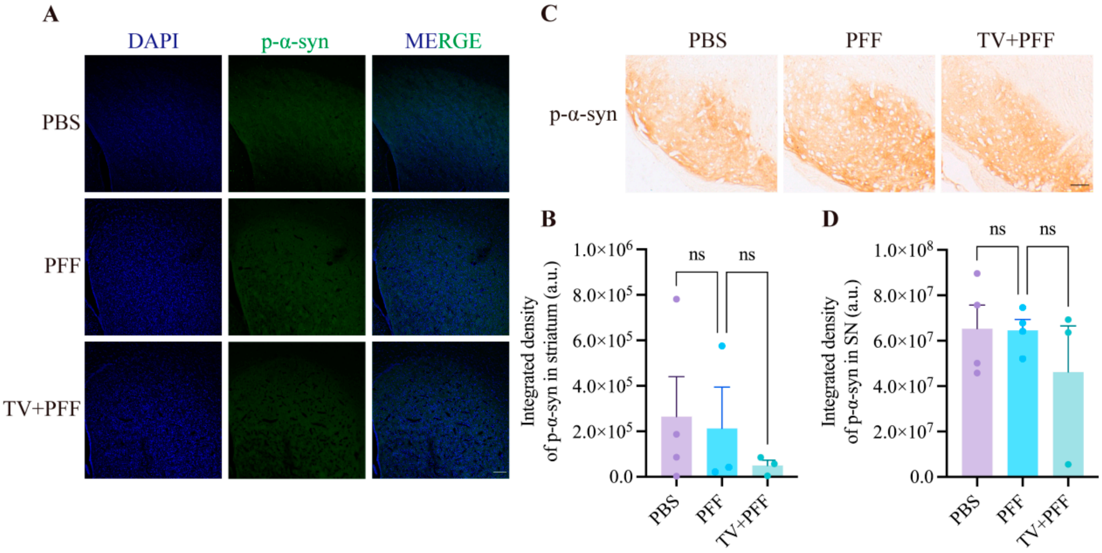

Figure S2. No detectable signal by immunofluorescence staining or immunohistochemistry staining in the striatum or SN of C57BL/6J mice. A. Representative immunofluorescence staining images of p-α-syn (green) and DAPI (blue) in striatum. Scale bar: 100 μm. B. Quantification of p-α-syn integrated density in striatum (n = 3-4 mice/group). C. Representative immunohistochemical staining images of p-α-syn in SN. Scale bar: 100 μm. D. Quantification of p-α-syn integrated density in SN (n = 3-4 mice/group). Data are presented as

mean ± SEM. Statistical analysis was performed using one-way ANOVA followed by Dunnett's test. ns, not significant. PBS group, mice receiving PBS injection into the duodenal muscular layer; PFF group, mice receiving α-syn PFF injection into the duodenal muscular layer; TV + PFF group, mice subjected to truncal vagotomy followed immediately by α-syn PFF injection.

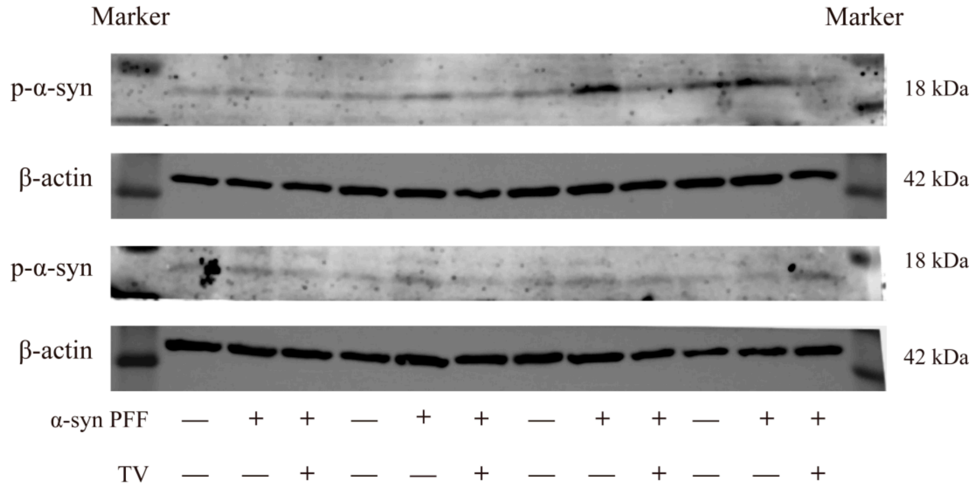

Figure S3. The original blots of p-α-syn expression in the striatum of C57BL/6J mice. These images correspond to the data quantified in Figure 3J. PBS group, mice receiving PBS injection into the duodenal muscular layer; PFF group, mice receiving α-syn PFF injection into the duodenal muscular layer; TV + PFF group, mice subjected to truncal vagotomy followed immediately by α-syn PFF injection.
